# Prognostic Features of the Tumor Immune Microenvironment in Glioma and Their Clinical Applications: Analysis of Multiple Cohorts

**DOI:** 10.3389/fimmu.2022.853074

**Published:** 2022-05-23

**Authors:** Chunlong Zhang, Yuxi Zhang, Guiyuan Tan, Wanqi Mi, Xiaoling Zhong, Yu Zhang, Ziyan Zhao, Feng Li, Yanjun Xu, Yunpeng Zhang

**Affiliations:** College of Bioinformatics Science and Technology, Harbin Medical University, Harbin, China

**Keywords:** glioma, immune microenvironment, prognosis, subpathway, multiple cohorts

## Abstract

Glioma is the most common malignant tumor of the central nervous system. Tumor purity is a source of important prognostic factor for glioma patients, showing the key roles of the microenvironment in glioma prognosis. In this study, we systematically screened functional characterization related to the tumor immune microenvironment and constructed a risk model named Glioma MicroEnvironment Functional Signature (GMEFS) based on eight cohorts. The prognostic value of the GMEFS model was also verified in another two glioma cohorts, glioblastoma (GBM) and low-grade glioma (LGG) cohorts, from The Cancer Genome Atlas (TCGA). Nomograms were established in the training and testing cohorts to validate the clinical use of this model. Furthermore, the relationships between the risk score, intrinsic molecular subtypes, tumor purity, and tumor-infiltrating immune cell abundance were also evaluated. Meanwhile, the performance of the GMEFS model in glioma formation and glioma recurrence was systematically analyzed based on 16 glioma cohorts from the Gene Expression Omnibus (GEO) database. Based on multiple-cohort integrated analysis, risk subpathway signatures were identified, and a drug–subpathway association network was further constructed to explore candidate therapy target regions. Three subpathways derived from Focal adhesion (path: 04510) were identified and contained known targets including platelet derived growth factor receptor alpha (PDGFRA), epidermal growth factor receptor (EGFR), and erb-b2 receptor tyrosine kinase 2 (ERBB2). In conclusion, the novel functional signatures identified in this study could serve as a robust prognostic biomarker, and this study provided a framework to identify candidate therapeutic target regions, which further guide glioma patients’ clinical decision.

## Introduction

Glioma is the most common malignant tumor of the central nervous system (CNS), accounting for 30% (80%) of all brain (malignant) tumors, which displayed the representative characteristics of strong genetic heterogeneity, high mortality, and chemotherapy resistance (1, 2). According to the World Health Organization (WHO) criteria, glioma tumors are histologically separated into Grade I through IV. Despite significant improvements in the glioma clinical treatment strategy, the median survival remains poor, particularly for those with glioblastoma (GBM, the grade IV of glioma). Patients with GBM exhibit a median survival of approximately 1 year and display poor responses to nearly all clinical therapies (3). Therefore, it is necessary to dissect the inner biological mechanism involved in glioma patients’ survival and identify a novel and robust predictive signature for clinical treatment guidance.

Glioma tissue included both cancer cells and non-transformed cells, which included predominantly resident microglia from the brain and circulating blood monocytes (macrophages), comprising 30%–50% of the cellular content of these tumors (4). The glioma purity was closely related to patient prognosis (5, 6), implying that the glioma immune microenvironment was involved in key glioma biology, including prognosis, recurrence, and treatment response. Recently, many immune-related signatures were identified for glioma prognostic analysis and functional exploration. A ferroptosis-related gene signature was identified and correlated with the tumor immune microenvironment status in the study of Zheng et al. (7), and this signature displayed a predictive performance for glioma survival. Cheng et al. (8) performed a multi-omics integrated analysis and identified an immune-related gene signature (ABCC3, PDPN, and INA) to predict the prognosis, immune infiltration status, and immunotherapy and chemotherapy response of LGG patients with epilepsy. Zhao et al. (9) performed a glioma immune subtype analysis based on 29 immune cell characterizations and defined three immune groups, named immune-H, immune-M, and immune-L. The redefined immune phenotypes were related to patient survival and contributed to the remodeling of the immunosuppressive microenvironment (9). However, in addition to immune cell infiltration, microenvironment features should also include tumor features, such as stromal remodeling, proliferation, and tumor procytokines, and consider the full range of immune-related characteristics. Therefore, it is necessary to characterize the glioma immune microenvironment based on sufficient samples from multiple cohorts, especially considering the immune cells surrounding the tumor, for further guiding the clinical prognosis.

In this study, by analyzing available glioma expression profiles with survival data of a total of 3,486 samples from eight cohorts, we utilized the Least Absolute Shrinkage and Selection Operator (LASSO) model to construct the Glioma MicroEnvironment Functional Signature (GMEFS) model, which consisted of 25 immune microenvironment signatures. To test the prognostic performance of GMEFS, we further obtained two independent glioma cohorts from The Cancer Genome Atlas (TCGA) database, and the nomogram was constructed for evaluating the prognostic performance. Meanwhile, the associations between GMEFS and glioma molecular subtypes, tumor purity, or stromal score, as well as immune cell infiltration, were explored. The GMEFS score was also evaluated in multiple-level brain tumor formation and glioma recurrence from a large number of glioma samples from multiple cohorts. Finally, we identified risk subpathways related to the GMEFS score and constructed a comprehensive drug–subpathway network for candidate target region screening, which provided important guidance for glioma clinical treatment.

## Materials and Methods

### Publicly Available Training and Testing Cohort Datasets

We searched the available mRNA expression profiles with prognosis information from several tumor resources, such as the Gene Expression Omnibus (GEO) database, Chinese Glioma Genome Atlas (CGGA) database, and Pan-Cancer Analysis of Whole Genomes (PCAWG) database for glioma prognostic microenvironment identification. For the datasets from the GEO database, the cohorts with at least 40 samples were considered and a total of six public glioma cohorts were downloaded. We also collected two glioma cohorts (LGG and GBM) from TCGA database and the multi-omics data including gene expression, methylation level, and copy number variations (CNVs) from the Human Glioma Cell Culture (HGCC) collection as the independent testing set. Finally, 6,920 brain samples from 27 cohorts were included in our study, and the total information was shown in **Table 1**.

**Table 1:** The information of all datasets used in this article.

Dataset	Sample	Survival time^c^	Usage in this study				
			Training	^d^Sur	^e^Inc	^f^Rec	Drug Network
GSE7696	84	19.25 ± 15.19	√		√		
GSE42670	56	20.88 ± 13.20	√				
GSE50021	45	12.26 ± 14.48	√		√		
GSE72951	110	10.88 ± 7.56	√				
GSE74187	60	19.15 ± 10.58	√				
GSE83300	50	19.07 ± 10.50	√				
CGGA	2,063	44.63 ± 43.07	√			√	
PCAWG	2,419	36.51 ± 42.26	√				
GBM	151	13.79 ± 12.88		√			
LGG	508	32.11 ± 31.98		√			
GSE4290	176				√		
GSE9385	55				√		
GSE15824	45				√		
GSE16011	284				√		
GSE22866	46				√		
GSE35493	21				√		
GSE42656	42				√		
GSE44971	58				√		
GSE50161	62				√		
GSE61335**^a^**	62				√		
GSE61335**^b^**	62				√		
GSE116520	42				√		
GSE60898	151					√	
GSE62153	43					√	
GSE98995	68					√	
GSE101113	56					√	
HGCC	101						√

^a^GPL19180, ^b^GPL19184, ^c^mean survival time ± SD, ^d^Survival verify, ^e^Incidence verify, ^f^Recurrence verify.

### Immune Microenvironment Signatures

We collected 175 immune microenvironment-related signatures from diverse literature for glioma prognostic signature identification. In detail, 28 signatures were obtained from the work of Bindea et al. (10), 11 signatures were obtained from the work of Wolf et al. (11), 24 cell signatures were obtained from the work of Miao et al. (12), 22 immune signatures were obtained from CIBERSORT (13), 29 immune microenvironment signatures were obtained from the work of Bagaev et al. (14), 40 signatures were obtained from Cellmarker database (15), 4 microglia signatures were obtained from the work of Sala Frigerio et al. (16), 8 brain immune cell signatures were obtained from scREAD database (17), and 9 microglia subtype signatures were obtained from the work of Olah et al. (18) More detailed information is listed in **Table S1**.

The datasets from GEO, CGGA, and PCAWG databases were treated as the training set, and the datasets from TCGA were treated as the testing set. Based on the 175 tumor microenvironment signatures, we firstly utilized the single sample Gene Set Enrichment Analysis (ssGSEA) method implemented in the R package to calculate the normalized enrichment score (NES) for each glioma sample from the training set (19). To remove the potential batch effects from different cohorts, we further used Combat function to form a merged NES matrix for microenvironment signature identification. Finally, the prognostic performance of this signature was tested using testing datasets.

### Construction of the Immune Microenvironment Risk Model

Based on the merged NES matrix, which consisted of 175 immune microenvironment signatures and 4,887 glioma samples, we firstly performed univariable Cox proportional hazards regression analysis using 3,486 samples with survival analysis. A set of 141 signatures was identified with a prognostic P-value <0.05. The detailed univariable Cox results, including the hazard ratio (HR) value, 95% CI, and P-values, of these microenvironment signatures were provided in **Table S2**. The LASSO Cox regression model (20) was used to find the most useful prognostic markers among the 141 immune microenvironment signatures in the training cohort by the R package glmnet. Ultimately, 25 immune microenvironment features with non-zero coefficients were selected through LASSO Cox regression model analysis, and the optimal lambda value was determined by 10-fold cross-validation. The multivariable Cox proportional hazards regression analysis was further performed for these 25 immune microenvironment signatures obtained from the LASSO analysis, and the corresponding coefficients are presented in **Table S3**. Finally, a novel glioma prognostic signature, GMEFS, was constructed by comprehensively considering the coefficients of these 25 immune signatures. The formula was provided as follows:


$$\text{GMEFS}=\sum_{\text{i}=1}^{25}\log(\text{H}{\text{R}}_{i})\times \text{NE}{\text{S}}_{\text{i}}$$

where HR_i_ is the HR and NES_i_ is the NES for the ith immune microenvironment signature.

### Functional Analysis and Differential Expression Analysis

The ESTIMATE score, tumor purity, and stromal and immune scores for each glioma sample were calculated by using ESTIMATE package in R with default parameters (21). The differential expression (DE) analyses for TCGA GBM and LGG cohorts were performed by the limma package in R (22). Functional enrichment analyses, including Gene Ontology (GO) and Kyoto Encyclopedia of Genes and Genomes (KEGG) pathway and subpathway analyses, were performed by using the clusterProfiler package in R (23), and the P-value was adjusted by the Benjamini and Hochberg method.

### Drug–Subpathway Network

Based on the HGCC resource (24), we obtained the drug IC50 information, as well as gene expression, methylation, and CNV data for each GBM cell line. Firstly, based on the median IC50 value as cutoff, we defined two cell line groups, high IC50 groups and low IC50 groups. Then, based on these two groups, we respectively identified drug-related genes according to gene expression level, methylation condition, and CNV data. For gene expression profiles, the T-test was used. For methylation and CNV data, the Wilcoxon rank sum test was used. The cutoff for DE analysis was set as adjusted P-value <0.05. Finally, we evaluated the associations between DE genes and GMEFS subpathways by using the hypergeometric test method. The result with P-value <0.05 was considered as a significant association. An integrated drug–subpathway network was constructed by considering the significant drug–subpathway associations shared by two omics results.

### Statistical Analysis

According to the GMEFS score, all glioma samples from both training and testing sets were classified into two groups based on the consistent cutoff as the training set. Then, the Kaplan–Meier (KM) curve and survival P-value calculated by the log-rank test were performed by using R survminer package. The glioma molecular subtypes (Classical-like, Codel, G-CIMP-high, G-CIMP-low, Mesenchymal-like, PA-like) of the glioma patients were obtained from a previous study (25). For both training and testing sets, a nomogram was formulated to provide a visualized risk prediction after each factor was assigned a score. A calibration plot was generated to assess the calibration ability of the nomogram (26). Nomograms and calibration plots were generated by using the rms package. The decision curve analysis (DCA) was performed by using dca package. All of the P-value results were considered statistically significant with P-values <0.05.

## Results

### Identification of Glioma Immune Microenvironment Functional Signatures for Prognostic Analysis

**Figure 1:**
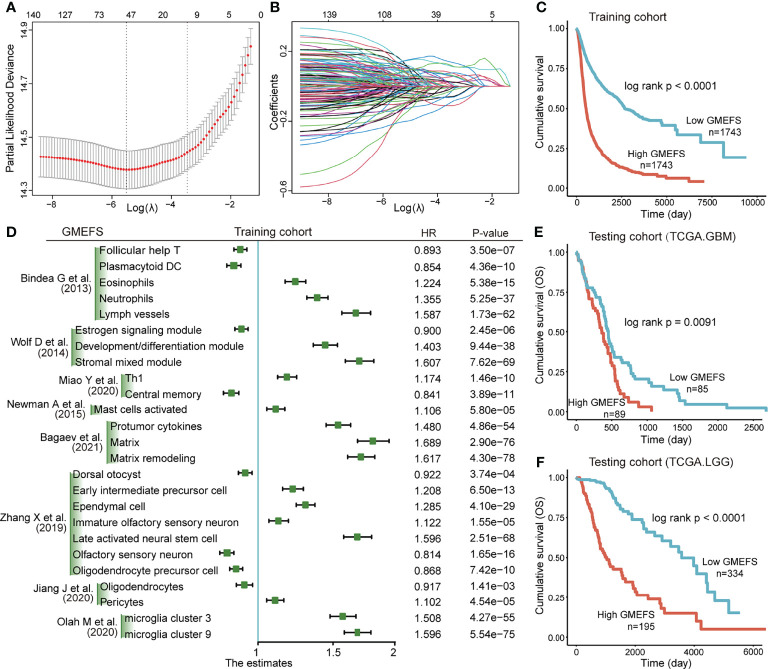
Construction and validation of the Glioma MicroEnvironment Functional Signature (GMEFS) model. **(A)** Partial likelihood deviance revealed by the Least Absolute Shrinkage and Selection Operator (LASSO) regression model in the 10-fold cross-validation. The vertical dotted lines were drawn at the optimal values by using the minimum and 1-SE criteria. **(B)** LASSO coefficient profiles of 25 selected immune cell signatures in the 10-fold cross-validation. **(C)** Kaplan–Meier estimate of the overall survival for the training cohorts. **(D)** The forest plot of the associations between the infiltrate levels of 25 immune cell signatures and overall survival in the training cohort. The HR, 95% CI, and P-value were determined by univariate Cox regression analysis. Kaplan–Meier estimate of the overall survival for two testing cohorts, The Cancer Genome Atlas-glioblastoma (TCGA-GBM) **(E)** and The Cancer Genome Atlas-low-grade glioma (TCGA-LGG) **(F)**, divided by the GMEFS model.

Based on the training set that consisted of a total of 8 glioma cohorts, we constructed a microenvironment-based prognostic model named GMEFS (see *Materials and Methods*). The GMEFS consisted of 25 glioma immune microenvironment signatures, and the detailed LASSO results of these 25 signatures were displayed in **Figures 1A, B**. We calculated the GMEFS score for each glioma patient in the training cohort and stratified the patients into high or low GMEFS groups according to the median cutoff. As shown in **Figure 1C**, the samples with a high GMEFS score had a significantly shorter overall survival than samples with a low GMEFS score in the training cohort (P < 0.0001; log-rank test). The associations between each of the 25 immune microenvironment signatures and clinical survival are also shown in **Figure 1D**. To further examine the robustness of the GMEFS model, two independent glioma cohorts, GBM and LGG datasets, were also obtained as testing sets for the prognosis analysis (see *Materials and Methods*). With the same formula, the samples from the testing set were stratified into high and low GMEFS groups by the cutoff value obtained from the entire training set. As shown in **Figures 1E, F**, the samples with a high GMEFS score also had significantly worse overall survival than those who displayed a low GMEFS score in both GBM and LGG cohorts (GBM P = 0.0091, LGG P < 0.0001; log-rank test). Meanwhile, survival analysis for progression-free survival (PFS) was also performed, and the predictive performance of GMEFS in GBM (**Supplementary Figure 1A**, P = 0.015) and LGG (**Sepplementary Figure 1B**, P < 0.0001) was confirmed. Similar results were observed in the entire TCGA cohort for both overall survival and PFS (**Supplementary Figures 1C, D**).

**Figure 2:**
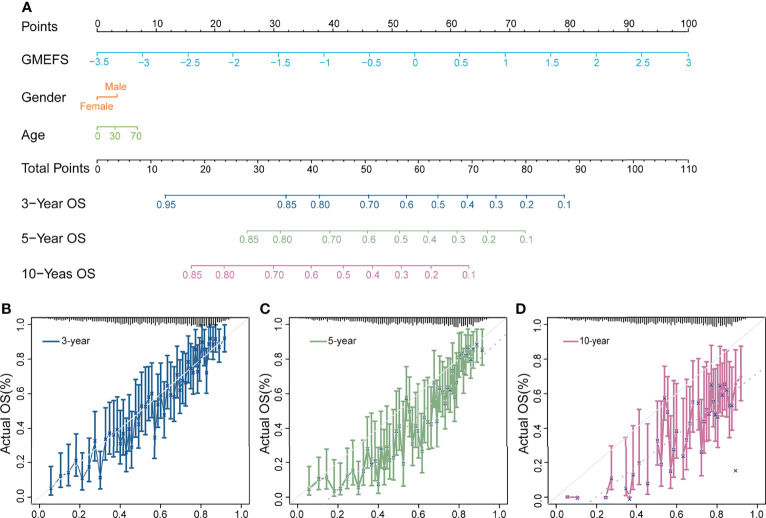
Nomogram developed for predicting the probability of 3-, 5-, and 10-year overall survival in the training cohort. **(A)** The nomogram was constructed in the training cohort, with the GMEFS and available clinical factors incorporated. Calibration plot of the nomogram in terms of agreement between the predicted and observed **(B)** 3-, **(C)** 5-, and **(D)** 10-year outcomes.

To further test the performance of predicting the glioma patient prognosis, a nomogram that integrated both the GMEFS and clinical factors (including gender and age) was constructed by using patients from the training set. Based on the nomogram results, a score can be calculated for a glioma patient for predicting the 3-, 5-, and 10-year overall survival for an individual, suggesting the power of GMEFS score in contributing the risk point (**Figure 2A**). The calibration curves for 3, 5, and 10 years of the training cohort were respectively illustrated in **Figures 2B–D**, showing the GMEFS’s performance in the glioma patient survival prediction.

### The Clinical and Functional Characterizations Between the Samples With High and Low GMEFS Scores

**Figure 3:**
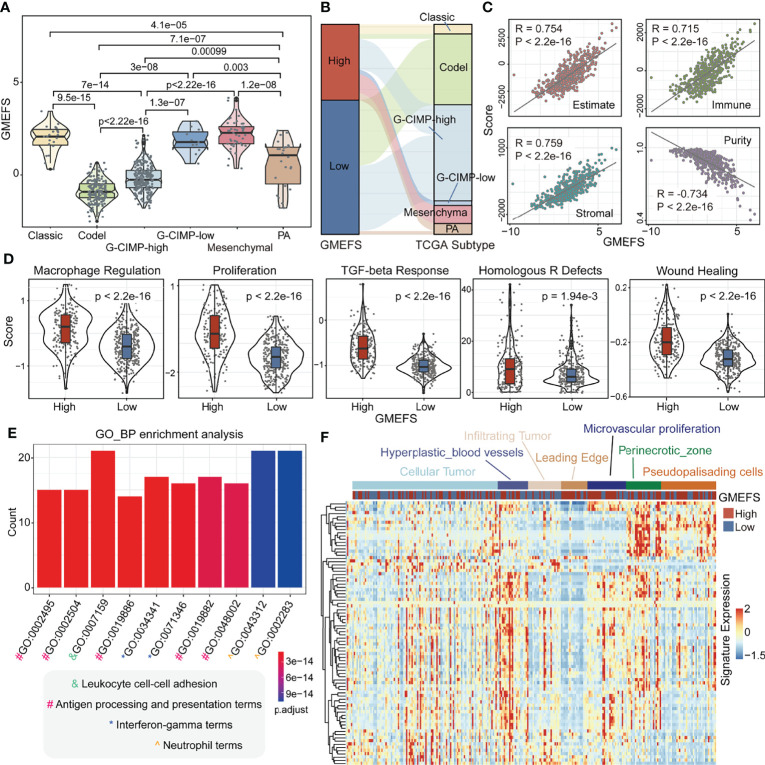
Clinical significance and functional analysis of the two GMEFS groups in TCGA-LGG. **(A)** Violin plot illustrating the distribution of the GMEFS score in different intrinsic molecular subtypes. **(B)** Sankey plot of the GMEFS values in subtypes with different intrinsic molecular subtypes. **(C)** Correlations between the GMEFS score, stromal score, immune score, ESTIMATE score, and tumor purity. **(D)** Comparison of Macrophage Regulation, Proliferation, TGF-beta Response, Homologous R Defects, Wound Heal between the high and low GMEFS groups. TCGA-LGG patients were classified into low and high GMEFS groups. Visualization of the top 10 enriched biological processes **(E)** by the upregulated differentially expressed genes (DEGs) in the high vs. low GMEFS groups. **(F)** Heatmap of the upregulated DEGs in the Ivy Glioblastoma Atlas Project (IGAP) dataset. Samples were ordered along the row by the structure regions.

To test the associations between GMEFS and molecular subtypes, we obtained the glioma subtype information (see *Materials and Methods*). As shown in **Figure 3A**, the samples from Classic-like, G-CIMP-low, and Mesenchymal-like subtypes displayed high GMEFS scores, whereas samples from the Codel subtype had a low GMEFS score, and the overall difference was significant. Moreover, an imbalance in terms of these five molecular subtypes within the two GMEFS group was observed (**Figure 3B**). Only small proportions of Codel subtype (6.5%) distributed in the high GMEFS group in contrast with 100% of Mesenchymal-like type and 100% of Classic-like type. Similar results were observed between molecular subtype and GBM GMEFS groups. Then, the correlations between tumor purity, stromal characterization, and GMEFS score were also explored. It was observed that the GMEFS score was positively correlated with the stromal score, immune score, and ESTIMATE score, whereas it was negatively correlated with tumor purity for TCGA LGG cohorts (**Figure 3C**), showing that the GMEFS score was a presentation of the immune microenvironment characterization.

Some potential factors that determine brain tumor immunogenicity, including macrophage regulation, homologous recombination deficient (HRD), TGF-beta response, and wound healing, were compared between the high GMEFS group and the low GMEFS group from TCGA LGG cohort (**Figure 3D**). The mean activity values for other factors of the high GMEFS group were significantly higher than those of the low GMEFS group. In addition, the samples with a higher GMEFS displayed a higher proliferation score than that of patients with a lower GMEFS, which was consistent with the prognostic performance of GMEFS. Overall, the differences in brain tumor immunogenicity between the GMEFS groups were significant, and the high GMEFS group had a relatively high immunogenicity. Next, we identified 83 upregulated genes in the high GMEFS group vs. the low GMEFS group by the cutoffs of log-fold change (FC) >1.5 and false discovery rate (FDR) <0.05. The GO biological process terms enriched by these genes included many immune-related processes, such as neutrophil activation, antigen processing and presentation, and neutrophil-mediated immunity (**Figure 3E**). Similar GO biological process terms and KEGG pathway results were illustrated in TCGA-GBM cohort (**Supplementary Figure 2**).

Furthermore, we quantified these 83 LGG gene signatures in data from the Ivy Glioblastoma Atlas Project (IGAP), which performed RNA sequencing (RNA-seq) on microdissections of glioma anatomical structures from hematoxylin and eosin (H&E) staining (Ivy Glioblastoma Atlas Project; http://glioblastoma.alleninstitute.org). The higher expression activity of these genes was enriched in samples from the hyperplastic blood vessels, microvascular proliferation, and perinecrotic zone. These gene signatures displayed a lower expression level in infiltrating tumor and leading edge (**Figure 3F**). To test the glioma association of 83 genes, we further obtained several glioma-related gene sets from a previous study (27). As shown in **Supplementary Figure 3A**, these genes displayed a significant overlap with GBM disease genes. These GBM genes were also enriched in the samples with a high GMEFS score compared to those with a low GMEFS score (**Supplementary Figure 3B**).

### Immune Cell Proportion Analyses for Samples With High and Low GMEFS Scores

**Figure 4:**
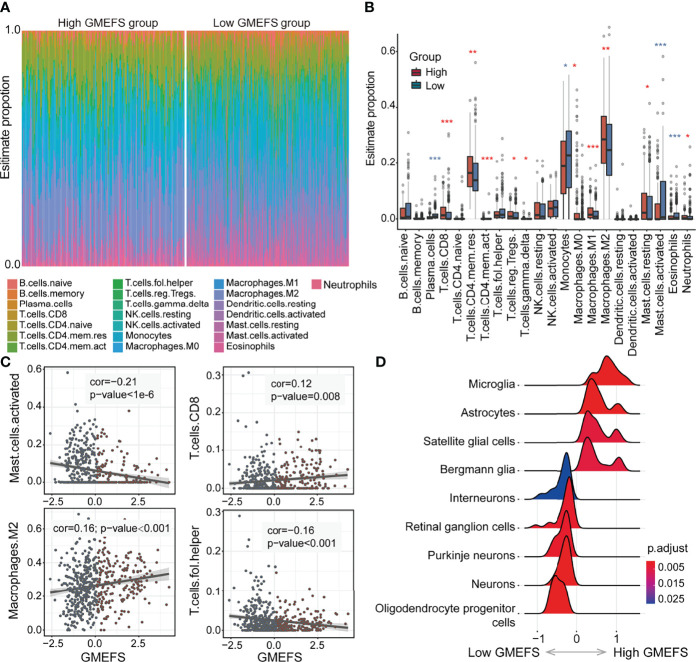
Immune cell proportion analyses of the two GMEFS groups in TCGA-LGG. **(A)** Relative proportions of immune infiltration for 22 signatures in the high and low GMEFS groups. **(B)** Boxplots illustrate the 22 immune cell proportions in TCGA-LGG. **(C)** The associations between the GMEFS score and infiltration score of activated mast cells, CD8 T cells, M2 macrophages, and T helper cells. **(D)** The GSEA analysis for cell markers from PanglaoDB database between the high and low GMEFS groups. *, 0.01<p; **, 0.001<p; ***, p-value<0.001.

To further explore the different immune cell proportions within the two GMEFS groups, we performed CIBERSORT analysis with 1,000 permutations for glioma cohorts (13). As shown in **Figures 4A, B**, the proportions of 22 common immune cell types of the two GMEFS groups of LGG were displayed. The samples from the low GMEFS group had significantly higher proportions of plasma cells, monocytes, activated mast cells, and eosinophils than those of samples from the high GMEFS group (P < 0.05). Correspondingly, the proportions of memory resting CD4 T cells, M2 macrophages, and CD8 T cells in the high GMEFS group were significantly higher than those in the low GMEFS group (P < 0.05). We further studied the correlation between the GMEFS score and the proportion of immune cell infiltration. The results revealed that CD8 T cells and M2 macrophages were positively correlated with the GMEFS, and activated mast cells and T helper cells were negatively correlated with GMEFS (**Figure 4C**). By comparing brain cell-type marker gene from the PanglaoDB database (28), we identified 9 cell types associated with the two GMEFS groups, including microglia, astrocytes, and oligodendrocyte progenitor cells (**Figure 4D**). Parenchymal microglia are the major component of myeloid cells involved in heterogeneous Central Neural System (CNS) immune microenvironment (29, 30), and microglia play important roles in the brain-related tumors, including glioma (31).

### The Involvement of GMEFS in Glioma Formation and Recurrence

**Figure 5:**
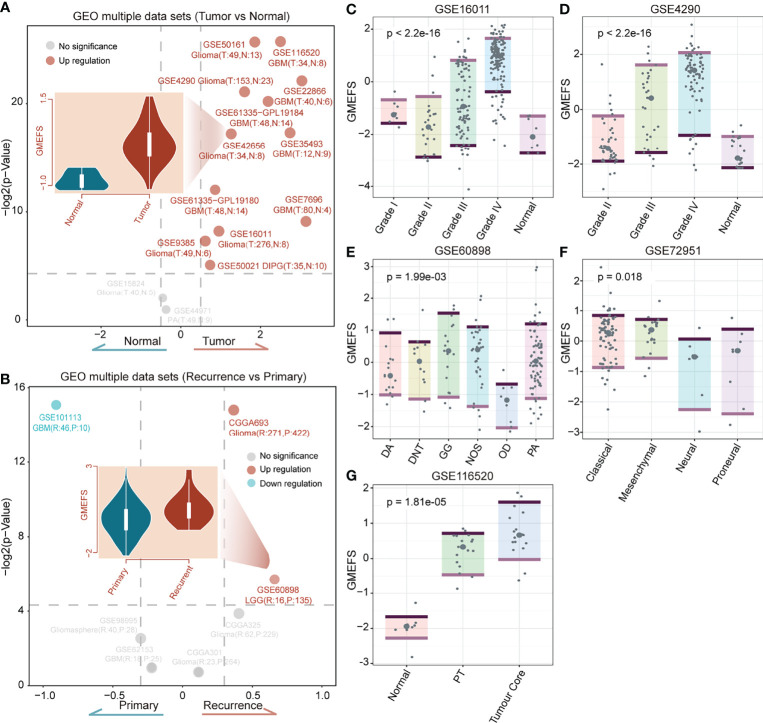
The confirmation of the GMEFS in glioma formation and recurrence. **(A)** Volcano plot of the GMEFS score between the two types of samples within each dataset for the tumor vs. normal conditions. **(B)** Volcano plot of GMEFS score between the two types of samples within each dataset for the recurrence vs. primary condition. Boxplot illustrating the distribution of the GMEFS score in different clinical information including clinical stage: **(C)** GSE16011, **(D)** GSE4290; molecular subtypes: **(E)** GSE60898, **(F)** GSE72951; and tumor regions: **(G)** GSE116520.

To test the performance of the GMEFS in glioma formation and recurrence, we further obtained the glioma datasets from the GEO database (**Table 1**). The GMEFS scores were calculated for each analytical sample, and the difference was evaluated between glioma and normal samples, as well as recurrence glioma and primary glioma samples. As shown in **Figure 5A**, among 14 independent glioma cohorts, the glioma samples displayed consistently higher GMEFS scores than those in normal samples in a total of 12 cohorts. These significant cohorts contained glioma samples (11/12) and Diffuse Intrinsic Pontine Glioma (DIPG) samples (1/12). In addition, within 2 of 7 glioma cohorts, the recurrence samples also displayed higher GMEFS scores than those in primary samples (**Figure 5B**). All these findings show that the GMEFS was not only a risk characterization for evaluating high-risk samples from low-risk samples but also could distinguish glioma samples from normal samples, considering the diagnosis index for glioma patients. As shown in **Figures 5C, D**, within two glioma cohorts with available stage information, we observed that the samples with a higher stage displayed a higher GMEFS score than samples with a lower stage, which was consistent with the prognostic performance of the GMEFS groups (GSE16011, P = 1.9e-08; GSE4290, P < 2.2e-16). Using these testing sets from the GEO, we further confirmed the GMEFS difference in histology subtypes and molecular subtypes, which was also consistent with previous results (GSE60898 and GSE72951 from **Figures 5E, F**, **Figure 3A**). As shown in GSE116520 (**Figure 5G**), the GMEFS score was also related to glioma region distribution; GMEFS was higher in core tumor tissue and lower in non-neoplastic brain tissue.

### Subpathway-Level Exploration Driven by GMEFS and Drug–Subpathway Network

**Figure 6:**
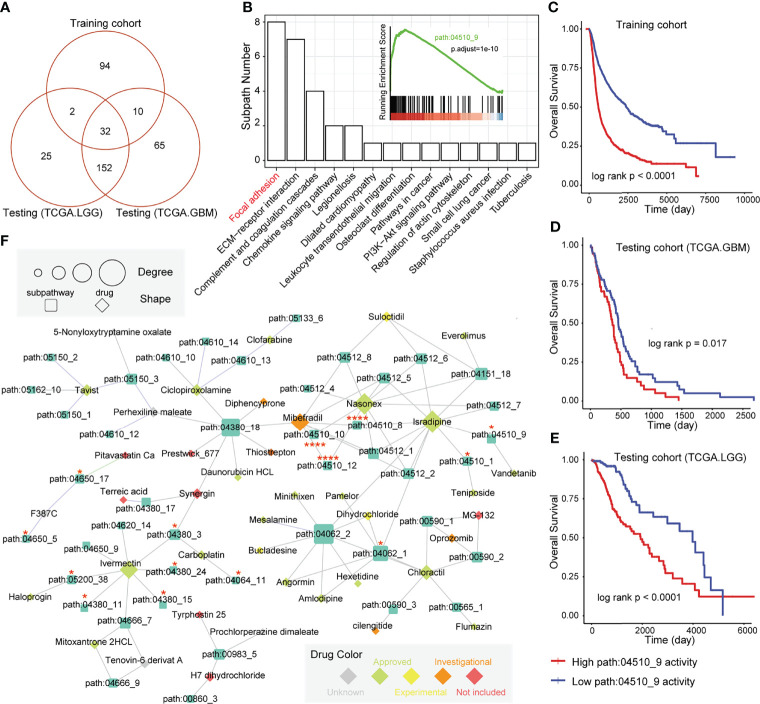
Subpathway-level functional exploration and drug–subpathway network. **(A)** Venn diagram of risk subpathways from training cohorts, testing TCGA-GBM cohorts, and TCGA-LGG cohorts. The risk subpathways were identified using the GSEA method based on the high and low GMEFS groups from the three cohorts. **(B)** The subpathway number statistics for 32 risk subpathways shared by the three cohorts. The Kaplan–Meier survival curves according to the activity value of one subpathway (path:04510_9) in the training cohort **(C)**, testing TCGA-GBM cohort **(D)**, and testing TCGA-LGG cohort **(E)**. **(F)** The drug subpathway network. The network shows the subpathways (squares) that could be targeted by drugs (diamonds) based on three levels of omics data from the HGCC database. The color of the drug indicates the five drug classes from the HGCC resource. The size of the subpathways or drugs increases with the degree that reflects the associations between the drugs and regulated subpathways.

In our previous studies (32, 33), we observed that the regions of the whole pathway, which is also named as subpathway, was closely related to disease formation and progression. The related results further confirmed that the subpathway displayed more advantages over the whole pathway with providing detailed biological information. We firstly obtained the subpathway list from subpathwayMiner R package with the default parameters (33). Based on the training, TCGA-GBM, and TCGA-LGG cohorts, we utilized the GSEA algorithm to identify the significantly enriched subpathways between the high GMEFS and low GMEFS groups. As shown in **Figures 6A, B**, a total of 32 risk subpathways were shared by three glioma cohorts. These risk subpathways were derived from Focal adhesion, ECM–receptor interaction, and pathways in cancer. Notably, a total of 8 subpathways derived from Focal adhesion were identified. Take one subpathway (path:04510_9) as an example, and the subpathway activity was calculated for the corresponding tumor samples. As shown in **Figures 6C–E**, the Focal adhesion subpathway could distinguish all samples into two groups with significant prognosis (Training cohort, p < 0.0001; TCGA-GBM, p = 0.017; TCGA-LGG, p < 0.0001). To explore the detailed associations between antineoplastic compounds and GMEFS subpathways shared by two cohorts, we constructed a multi-omic integrated network based on HGCC resource (see *Materials and Methods*). As shown in **Figure 6F**, many subpathways were targeted by many candidate drugs, such as path: 04062_2 from chemokine signaling pathway and path: 04510_9 from focal adhesion. The subpathway 04062_2 was targeted by many approved molecules, such as mesalamine, pamelor, angormin, and amlodipine. Some subpathways from the same whole pathway (path: 04510) were commonly targeted by some molecules, such as etoposide, paludrine, and suloctidil. Notably, as an experimental drug, suloctidil was closely related to many risk subpathways. Furthermore, we obtained several drug target genes for glioma collected from a recent study (34). We observed that many subpathways within networks contained known drug targets. Notably, three subpathways (path: 04510_8, _10, and _12) from Focal adhesion contained four target genes, including PDGFRA, EGFR, and SRC. The systematically reconstructed drug–subpathway network provided a novel framework for identifying subpathway regions as candidate therapy targets and screening-approved or experimental molecules as novel drugs for clinical use.

## Discussion

In this study, the relative quantitative infiltrate levels of 175 immune microenvironment signatures in a total of 4,887glioma patients from multiple cohorts were estimated, and a novel prognostic model (GMEFS) consisting of functional signatures was constructed. To test the predictive performance of the GMEFS, we further obtained independent glioma cohorts from TCGA. In addition, many other glioma cohorts from the GEO database were also obtained to analyze the different GMEFS scores between brain tumor and normal samples. Data from one glioma cell line from HGCC were utilized to construct a drug-related network. In a word, a total of 6,920 brain samples were utilized in this study to comprehensively identify and explore the glioma immune microenvironment characterization for prognosis analysis.

For the input in LASSO-Cox regression analysis, we have collected as many tumor-related microenvironment signatures as possible, including the functional signatures from the study of Bagaev et al. (14). A total of 175 immune microenvironment signatures were collected from diverse resources in this study. The 25 functional signatures in the GMEFS included 17 risk signatures with HR > 1 and 8 protective signatures with HR < 1. Among these 25 immune microenvironment signatures, some signatures were specific for the brain tissue, such as oligodendrocytes and pericytes from the study of Jiang et al. (17) and microglia clusters from the study of Olah et al. (18). Also, some neuron-related signatures were included in the GMEFS, such as Immature olfactory sensory neuron and late activated neural stem cell (as risk factors) and Olfactory sensory neuron (as protective factor). Moreover, some tumor characterizations were also included, such as protumor cytokines, matrix, and matrix remodeling from the study of Bagaev et al. (14), and all of these signatures displayed risk distribution in the GMEFS model. From another tumor study of Wolf et al. (11), two module signatures that the authors defined, Development/differentiation module and Stromal mixed module, were also identified.

Recently, a large scale of bioinformatics studies utilized public data resources to identify glioma prognostic signatures, including gene signatures (35–40), lncRNA signature (41), and gene-set signature (42, 43). The summary of all of these prognostic studies and our study was displayed in **Table S4**. Most of the previous studies utilized less than 6 cohorts to identity and validate the prognostic model, and the most frequent training sets were from TCGA or CGGA. Lin et al. (35) constructed a 5 gene-based prognostic model that was derived from hypoxia function to predict the glioma survival. The glioma cohorts from CGGA were utilized for model construction (35). Tan et al. (38) constructed a 6-gene risk model based on glioma samples from TCGA and validated it using CGGA samples. Some other studies performed the survival analysis based on some key genes or gene sets without identifying signature procedures (39, 42). Regarding both the construction and validation of the prognostic model, an adequate number of the training set and testing set were necessary for a robust risk model analysis. In our GMEFS construction, we utilized a total of 3,486 samples with survival information from 8 glioma cohorts to perform signature identification, and a total of 27 multiple cohorts were included in our analysis.

Based on TCGA-LGG cohort with available treatment information, we further observed that the GMEFS was significantly decreased in patients with complete or partial response when compared with those with stable or progressive disease. The effectiveness of the GMEFS value in predicting the response of cancer patients to immunotherapy was also verified (**Supplementary Figure 4**). TCGA-GBM, which contained a limited number of samples with available response information, was not included in this analysis.

Some limitations were also displayed in this study. Firstly, the signatures involved in the GMEFS were an independent functional set, and the complex interactions between tumor cells and immune cells were not explored. Secondly, the key gene or protein molecules underlying the GMEFS should further be identified, or the key signatures of the GMEFS should be further mined. However, it also displayed advantages over other studies that a total of more than 6,000 brain samples were utilized for constructing and verifying the prognostic and distinguishing glioma sample performance. Meanwhile, we developed a novel framework to identify functional signatures and explore drug–subpathway associations for glioma treatment guidance.

## Data Availability Statement

The datasets presented in this study can be found in online repositories. The names of the repository/repositories and accession number(s) can be found in the article/**Supplementary Material**.

## Author Contributions

CZ, WM, and YXZ analyzed and interpreted the data. CZ, GT, YX, and FL performed the bioinformatics analyses. YZ, XZ, ZZ, GT, and YPZ performed the biological evaluation. CZ, YX, and YPZ wrote the article. All authors read and approved the final article.

## Funding

This work was supported by the National Natural Science Foundation of China (Grant Nos. 62172131 and 62101164).

## Conflict of Interest

The authors declare that the research was conducted in the absence of any commercial or financial relationships that could be construed as a potential conflict of interest.

## Publisher’s Note

All claims expressed in this article are solely those of the authors and do not necessarily represent those of their affiliated organizations, or those of the publisher, the editors and the reviewers. Any product that may be evaluated in this article, or claim that may be made by its manufacturer, is not guaranteed or endorsed by the publisher.

## Supplementary Material

Supplementary Figure 1 Kaplan-Meier estimate of the overall survival of the TCGA-GBM (PFS)(A), TCGA-LGG (PFS) **(B)**, TCGA-GBM&LGG (OS) **(C)** and TCGA-GBM&LGG (PFS) **(D)** divided by two GMEFS model.

Supplementary Figure 2 TCGA GBM patients were classified into high and low GMEFS groups. Visualization of the top 10 enriched biological processes **(A)** and the top 10 enriched KEGG pathways **(B)** by the DEGs in the high versus low GMEFS groups.

Supplementary Figure 3
**(A)** the venn plot between DEGs from TCGA LGG high GMEFS and low GMEFS groups, and glioma related gene set. **(B)** The GSEA plot for GBM disease genes in the all rank genes by two GMEFS groups.

Supplementary Figure 4 The associations between GMEFS groups and therapy response in the TCGA LGG cohorts. **(A)** Boxplot illustrating the distribution of GMEFS for patients with different therapy responses. Significance was determined by the Wilcoxon test. **(B)** Bar graph illustrating the numbers of clinical responses to therapy in the high and low GMEFS groups. **(C)** Waterfall plot illustrating the distribution of GMEFS for patients with different therapy responses.
