# Supplementary figures and images for: Prognostic Features of the Tumor Immune Microenvironment in Glioma and Their Clinical Applications: Analysis of Multiple Cohorts
Source: Front Immunol. 2022 May 23;13:853074. doi: 10.3389/fimmu.2022.853074 (PMC9168240; doi:10.3389/fimmu.2022.853074)

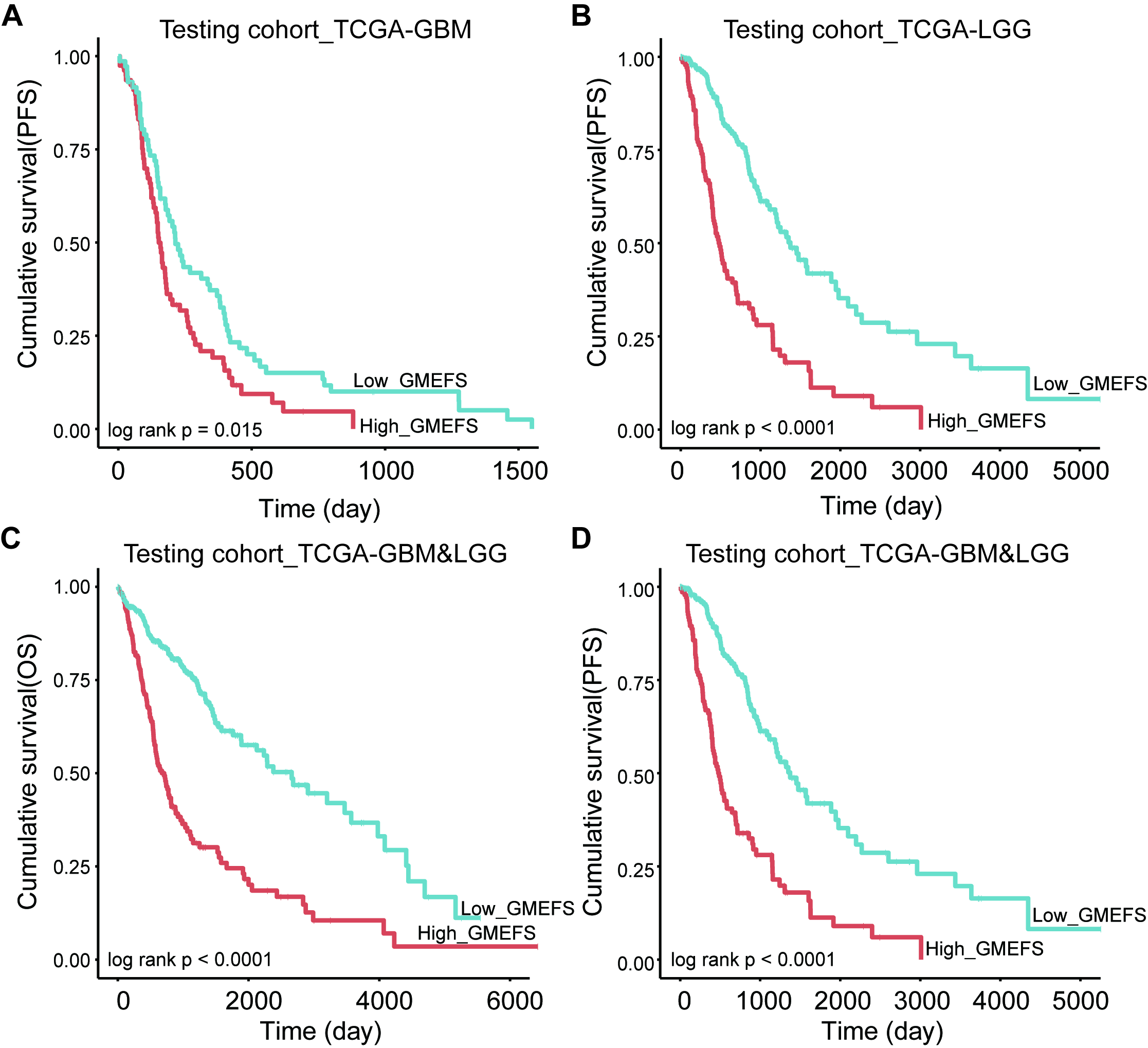

Supplement: Supplementary Figure 1 — Kaplan-Meier estimate of the overall survival of the TCGA-GBM (PFS)(A), TCGA-LGG (PFS) (B), TCGA-GBM&LGG (OS) (C) and TCGA-GBM&LGG (PFS) (D) divided by two GMEFS model. [file Image_1.tif]

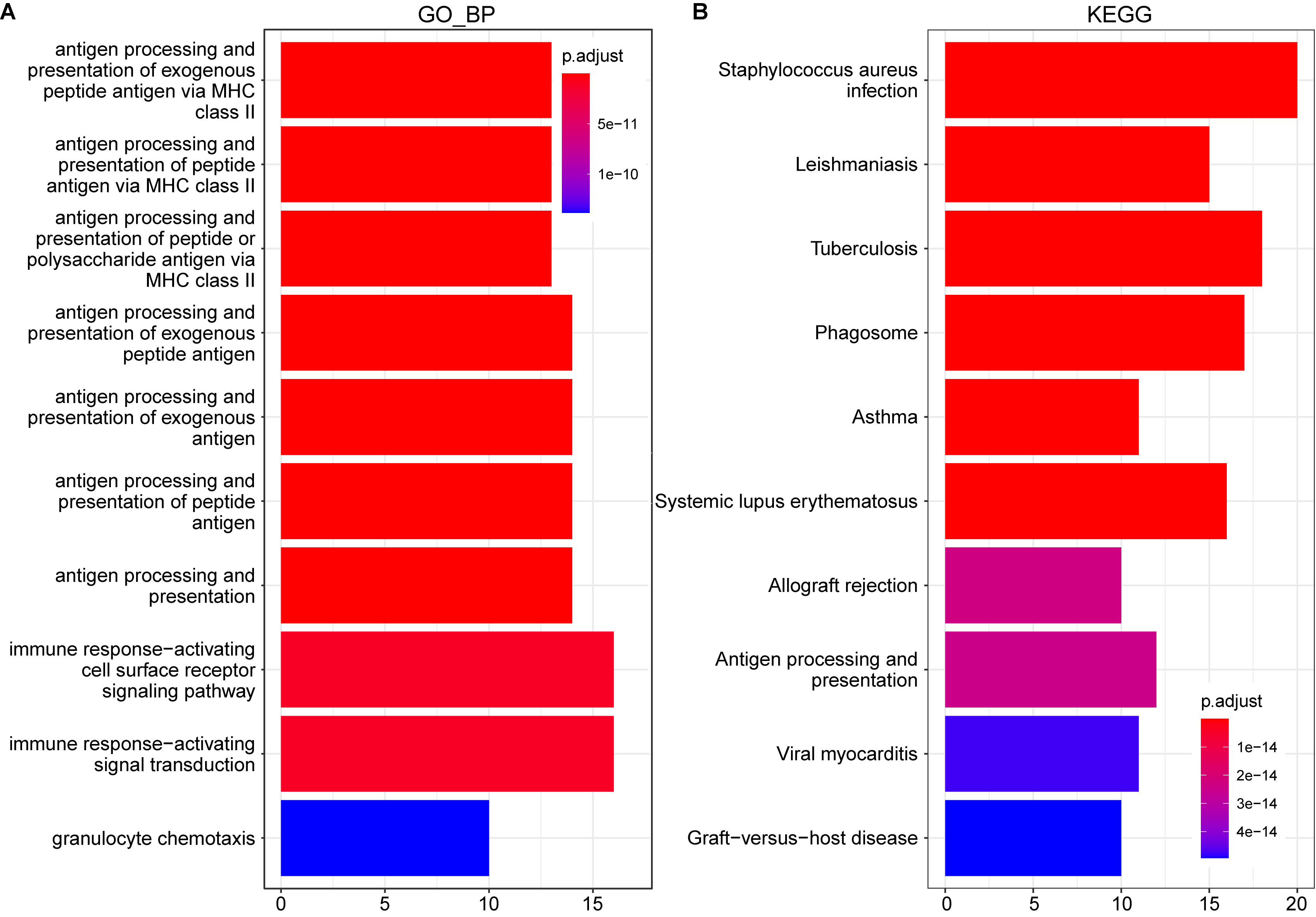

Supplement: Supplementary Figure 2 — TCGA GBM patients were classified into high and low GMEFS groups. Visualization of the top 10 enriched biological processes (A) and the top 10 enriched KEGG pathways (B) by the DEGs in the high versus low GMEFS groups. [file Image_2.tif]

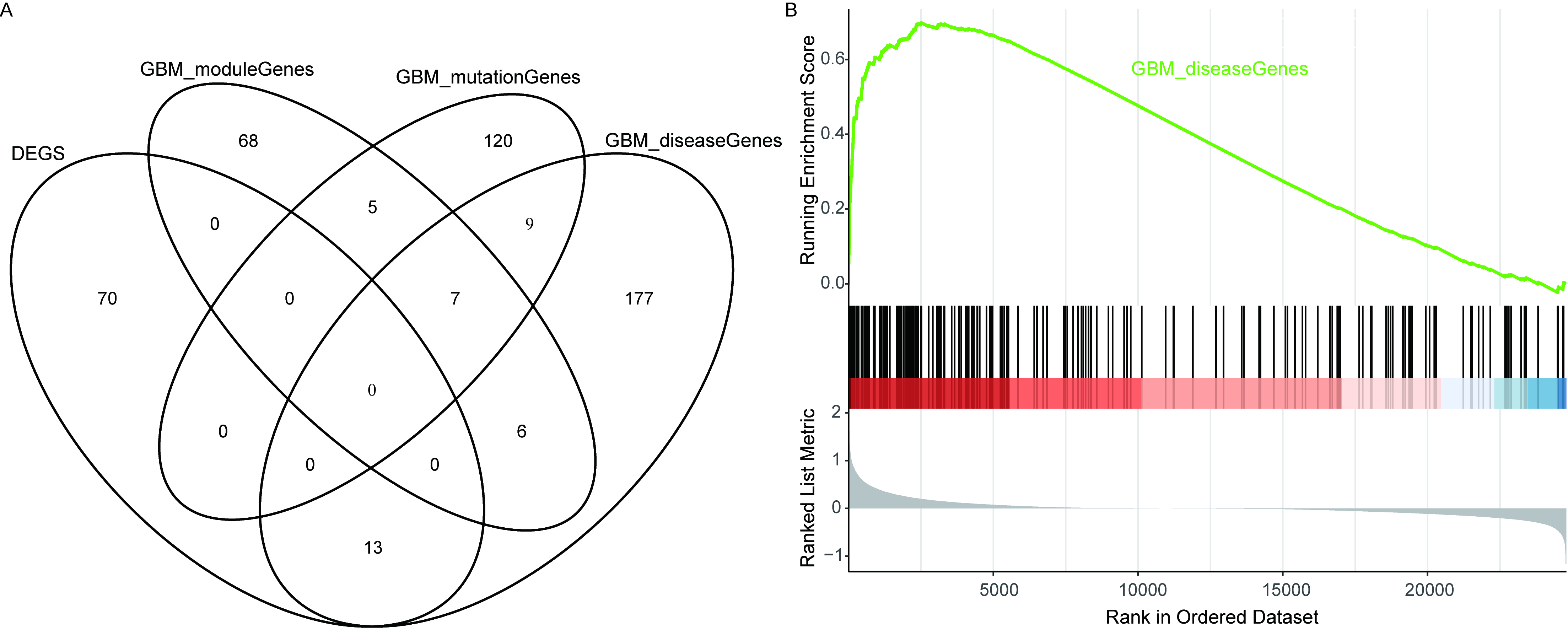

Supplement: Supplementary Figure 3 — (A) the venn plot between DEGs from TCGA LGG high GMEFS and low GMEFS groups, and glioma related gene set. (B) The GSEA plot for GBM disease genes in the all rank genes by two GMEFS groups. [file Image_3.tif]

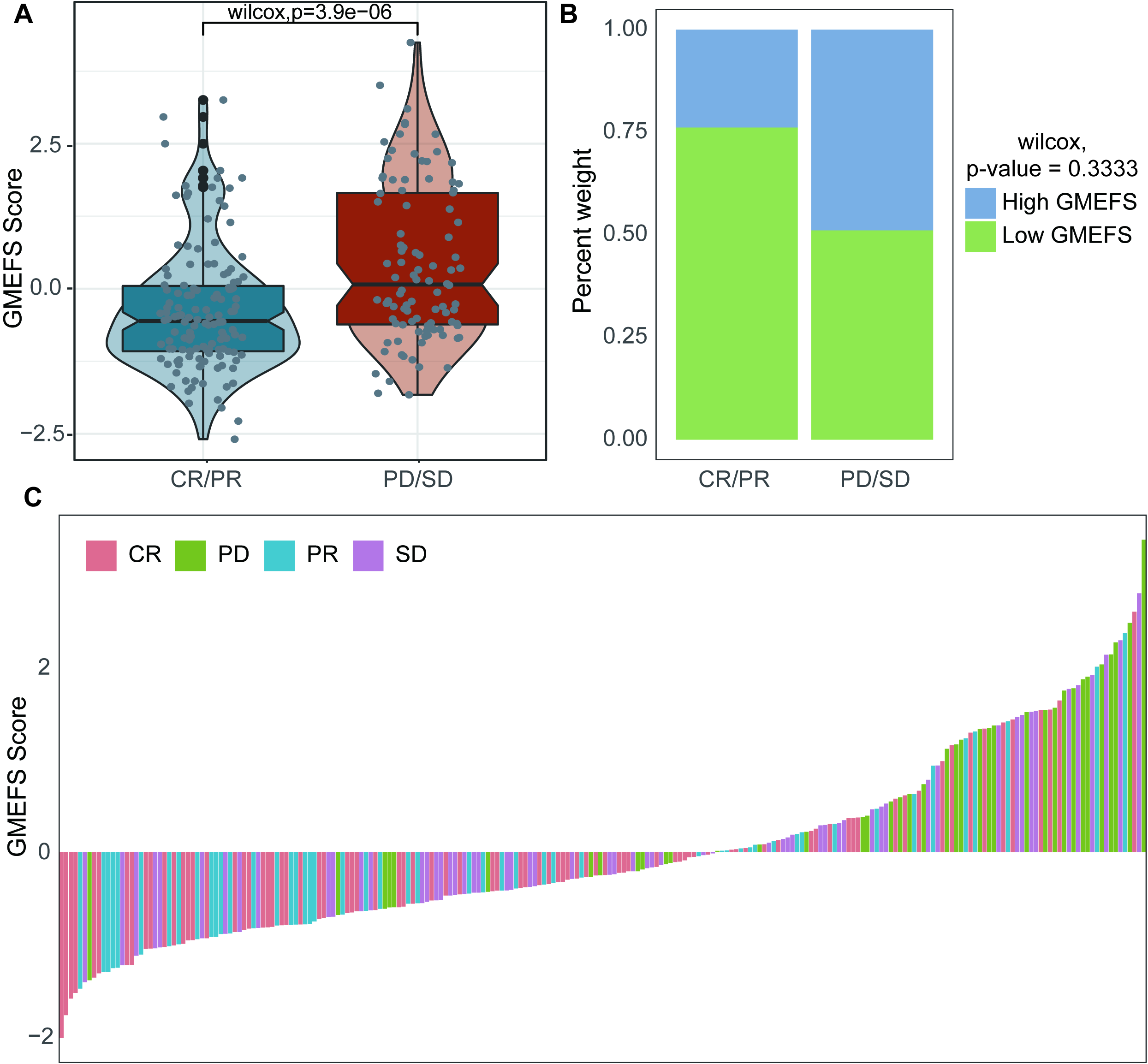

Supplement: Supplementary Figure 4 — The associations between GMEFS groups and therapy response in the TCGA LGG cohorts. (A) Boxplot illustrating the distribution of GMEFS for patients with different therapy responses. Significance was determined by the Wilcoxon test. (B) Bar graph illustrating the numbers of clinical responses to therapy in the high and low GMEFS groups. (C) Waterfall plot illustrating the distribution of GMEFS for patients with different therapy responses. [file Image_4.tif]
